# Zero-Lag Synchronization Despite Inhomogeneities in a Relay System

**DOI:** 10.1371/journal.pone.0112688

**Published:** 2014-12-08

**Authors:** Zahra Ghasemi Esfahani, Alireza Valizadeh

**Affiliations:** Institute for Advanced Studies in Basic Sciences, Zanjan, Iran; University of Maribor, Slovenia

## Abstract

A novel proposal for the zero-lag synchronization of the delayed coupled neurons, is to connect them indirectly via a third relay neuron. In this study, we develop a Poincaré map to investigate the robustness of the synchrony in such a relay system against inhomogeneity in the neurons and synaptic parameters. We show that when the inhomogeneity does not violate the symmetry of the system, synchrony is maintained and in some cases inhomogeneity enhances synchrony. On the other hand if the inhomogeneity breaks the symmetry of the system, zero lag synchrony can not be preserved. In this case we give analytical results for the phase lag of the spiking of the neurons in the stable state.

## Introduction

Throughout the cortex, the spiking activity of groups of cells exhibits various patterns of synchrony, during both spontaneous activity and under sensory stimulation [1]–[7]. Synchronous firing of neurons has received much attention in relation to the generation of brain rhythms and information processing at various aspects in the neuronal systems, such as selective attention and the binding problem [8]–[12]. Synchronized networks have a higher impact on their target networks, and the entrainment of a target network establishes an exclusive neuronal communication link [13]. It has been hypothesized that a dynamically changing coherent activity pattern may ride on top of the anatomical structure to provide flexible neuronal communication pathways [14]–[16].

Synchrony generation by networks of interconnected neurons is a subject of many theoretical and numerical studies [13], [17]–[24]. The mechanism of these phenomena has been subject of controversial debate in a more general context; beyond its functional relevance, the zero time lag synchrony among such distant neuronal ensembles must be established by mechanisms that are able to compensate for the delays involved in the neuronal communication [25]–[27].

A pair of neurons could either synchronize via direct connection or as a results of common inputs and it is quite probable that a variety of mechanisms are responsible for bringing synchrony at different levels (distinguishing for example, among local and long-distance synchrony) and different cerebral structures. Early studies on the synchronization of neurons with delayed connections showed that excitatory connections do not readily synchronize the neurons and in fact they usually lead to antiphase firing [28]–[30]. An almost complete framework has been eventually developed later, revealing the role of the phase response curve (PRC) in the synchronization of delayed coupled neurons [31]–[33]. PRC keeps track of how much an input advances or delays the next spike in an oscillatory neuron depending upon where in the cycle the input is applied [34]–[36] and is determined by the type of bifurcation which results in repetitive firing of the neuron [34], [35], [37], but see [38].

Synchronization is also affected by the configuration through which the neurons interact. Fischer et al. [39] introduced a novel mechanism of synchronization via dynamical relaying, followed by several other studies [40]–[42]. It has been shown that two distant neuronal populations are able to synchronize at zero time lag if a third element acts as a relay between them. This mechanism has proven to be remarkably robust for a broad range of conduction delays and cell types [43]. Biological relevance of the concept justified by proposing thalamus and hippocampus as the pivotal regions generating isochronal synchronization between distant cortical areas by means of the dynamical relaying mechanism [44]. Interestingly, connectivity studies in primate cortex have identified the relay pattern as the most frequently repeated motif at the level of corticocortical connections in the visual cortex, signaling the functional relevance of this topology in the cortical networks [45]–[49]. Synchrony induced by the relay configuration is based on the symmetric redistribution of incoming signals by the relay between the two outer neurons [50]. This symmetry needs both the equal parameters of the outer neurons and the symmetric links. In a minimal model, the neurons are characterized by their firing rates, and the links by the synaptic strengths and the delay times. All of these parameters bear substantial inhomogeneity in the brain networks [36], [51]–[53] and it is quite reasonable to investigate how such inhomogeneities can affect synchrony properties of the relay system. In this study we extensively explore the role of inhomogeneity in the neurons' and links' parameters on the synchrony of the outer neurons in the relay system. We use phase reduction approximation and the Poincare maps to derive analytic results for the stability of inphase (or near inphase) solutions in the presence of inhomogeneity. The role of firing rate of neurons, synaptic strengths and the transmission delay times is investigated. As the main outcome of the study, we show that symmetric inhomogeneities (those which preserve symmetry) have negligible disruptive effect on the synchrony and in some cases they can even improve it. On the other hand, we show that the relay system is vulnerable to the inhomogeneities which violate symmetry of the system. The analytic results are supported by the numerical experiments on the conductance based neuronal models.

## Methods

In our simulation we use Hodgkin-Huxley (HH) model described by a set of four variables 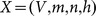, where 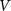 is the membrane potential, 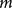 and 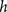 the activation and inactivation variables of the sodium current and 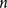 the activation variable of the potassium current. The corresponding equations of the motion read
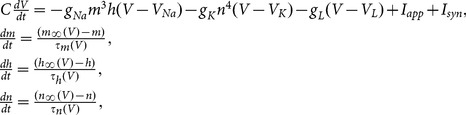
(1)where 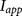 is the external current. The parameters 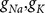 and 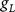 are corresponding reversal potentials and 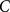 is capacitance per surface unit. We use the typical values the parameters as follows: 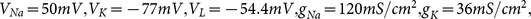 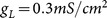 and 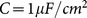. The functions 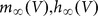 and 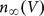 and the characteristic times (in milliseconds) 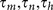 are given by: 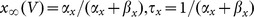 with 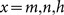 and 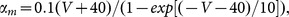 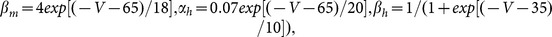 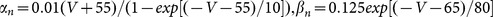. Let us recall that for small values of 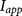 the system reaches a stable fixed point (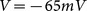 for 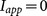). The transition from resting to spiking regime is mediated by a subcritical Hopf bifurcation at a critical value of input current 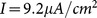. The synaptic current 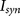 is modeled by 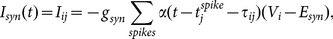
(2)where the 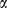 function 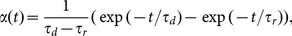
(3)shows postsynaptic conductance time course after each spiking of presynaptic neuron at time 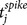. The time delay 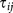 is the time needed for transmission of the signal from pre- to postsynaptic neuron, in this case from 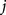
th to 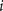
th neuron. The reversal potential of the synapse is 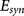 and 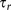 and 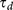 determine the rise time and decay time of the synaptic response, respectively. We set 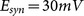 to model excitatory synapses, and 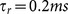 and 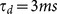.

### Phase-reduced models

Transition from resting to repetitive firing for HH neuron is mediated by a subcritical Hopf bifurcation [54]. With a suprathreshold constant current, HH model has an stable limit whose dynamics around the limit cycle can be well-approximated by the phase reduction method [54].

Assuming a network of coupled limit cycle oscillators described by 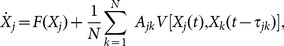
(4)where 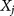 is the state of the 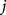
th oscillator, F(X) is the baseline vector field which describes single oscillator dynamics. A is the adjacency matrix of the network and V determines a coupling function. We assume the isolated neural oscillator has a stable limit cycle with period 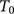, then a scalar phase variable 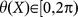 can be defined for all X in some neighborhood of the attracting limit cycle whose evolution is deduced from the chain rule 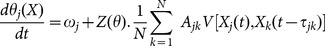
(5)


Using the phase sensitivity of each oscillator 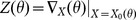 and droping the error term of 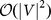, the dynamics of the system can be reduced to the phase equation 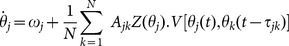
(6)which is valid in the attracting neighborhood of the limit cycle. Under the assumption that 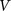 has nonzero components in just the voltage direction, we can define the phase sensitivity of each oscillator 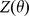, as the normalized phase response curve (PRC) as follows [54].

Suppose the oscillation period is 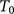. A brief stimulus is applied to the voltage variable in different times between two successive spikes of the neuron. This leads to a change in the time of the next spike. For small perturbation, PRC is defined as 
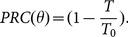
(7)


The phase sensitivity is the PRC divided by amplitude of small perturbation 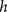 [55]. Since the PRC and phase sensitivity are linearly dependent in the small 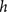 limit, we have used them equally when their functional form was intended. In our analytical studies, the spiking neurons are approximated with phase oscillators and the synaptic currents by pulsatile stimuli. Then the model reduces to 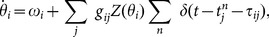
(8)where 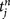 is the 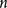
th spike time of 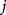 oscillator, which is presynaptic neuron, to the oscillator 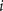 as the post synaptic neuron and, 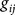 is the coupling strength from neuron 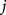 to 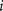.

In all the simulations, initial values for the dynamical variables are chosen from a uniform distrbution in the appropriate range for each variable. The results are recorded after discarding 50 periods to ensure the results are not affected by the transient dynamics.

## Results

### Two reciprocally coupled neurons

**Figure 1:**
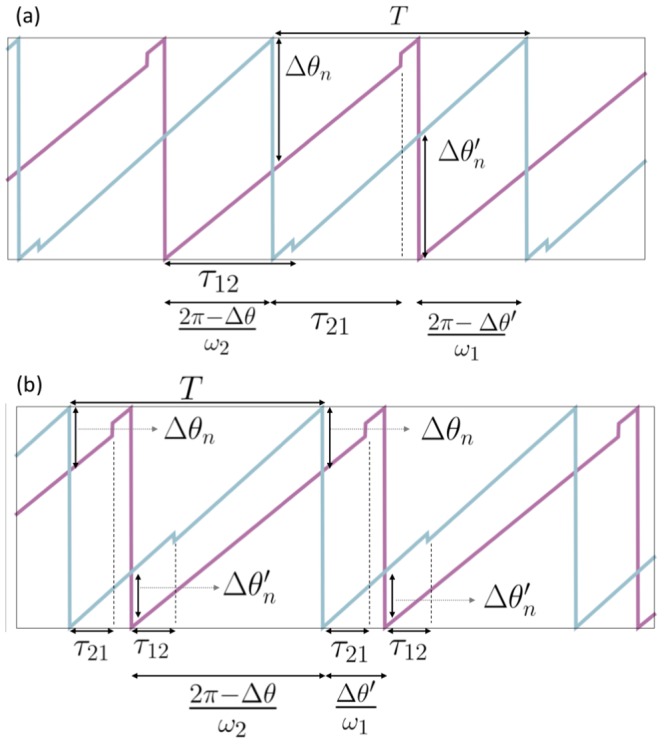
Time evolution of coupled phase oscillators. (a) Time evolution of two bi-directionally coupled phase oscillators for 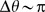. (b) Time evolution of two bi-directionally coupled phase oscillators for 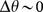. 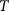 is the period of the oscillators in the phase-locked state and 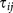 is the delay time from pre-synaptic neuron 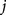 to post-synaptic neuron 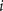. 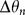 (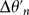) is the phase difference (modulo 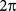) of two neurons at the 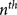 spiking time of first (second) neuron.

In this section we develop a map to analytically study the phase-locked state of two neurons connected through reciprocal delayed pulsatile couplings. The map and the formula presented here are complementary to the recent study [56] and give more precise results for a wider range of parameters (see below). To construct the map we record the phase of the neuron 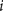 at the time of 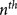 spike of the neuron 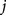, 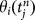. In a steady 1∶1 locked mode, depending on the phase lag of spiking of two neurons and the delay, two different situations can be observed (depicted in Fig. 1). In the first case (case 1) between every two spikes of the two neurons, both the neurons or non of them receive synaptic stimulations (Fig. 1a). In the other case (case 2) between every two spikes of the neurons only one of the neurons experiences an incident synaptic stimulation (Fig. 1b). In both cases we have (see Fig. 1): 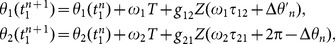
(9)where 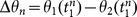 (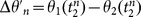) is the phase difference of two neurons at the 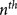 spiking time of first (second) neuron and will be used as the phase lag of the neurons in locked states. Assuming 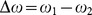, subtraction of the above equations gives 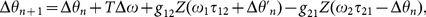
(10)where the period (inter-spike interval) in the locked state, 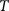, can be derived from 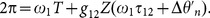
(11)


The locked state is characterized by the fixed points of Eq. 10. For case 1 there is another relation between the phase differences, 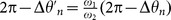 (see Fig. 1a). This is the main correction on the model introduced in previous study in which it has been assumed 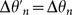 which is correct for 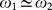 (see Eqs 8–10 in [56]). Setting 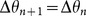 in Eq. 10 and eliminating period 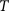 for case 1 gives 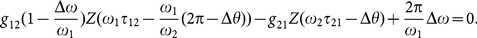
(12)


Given the functional form of phase sensitivity 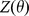, this implicit equation gives the phase lag in the locked state as a function of the parameters 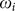, 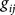 and 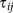.

For the second case, succession of the spikes of the neurons and the incident synaptic pulses is similar to what shown in Fig. 1b. In this case fixed points of the map Eq. 10 can be found from 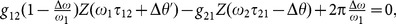
(13)where 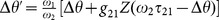 and 
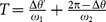
 which can be deduced from Fig. 1b.

Linearizing Eq. 10 around the fixed points gives the stability condition for the solutions 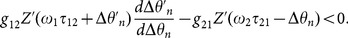
(14)where a prime denotes the derivative with respect to 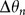.

**Figure 2:**
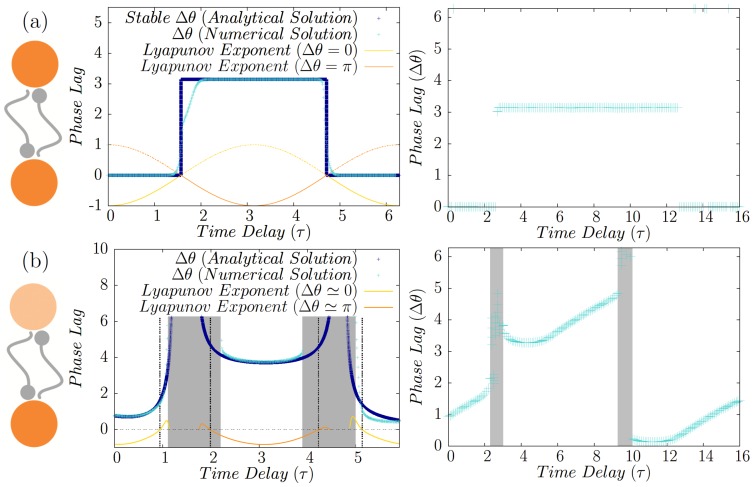
Homogeneous and inhomogeneous system of directly coupled neurons. (a) Synchronized states of two directly coupled identical type-II neurons. Depending on the delay time inphase or antiphase modes are stable with negative corresponding Lyapunov exponent which are shown for both the states (left panel). Phase difference (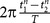) of firing of two identical Hodgkin-Huxely neurons is plotted in the right panel. (b) Synchronized states of two directly coupled neurons in presence of frequency mismatch. Numerical solutions (Cyan) affirm the validity of the analytical results obtained from linear approximation (Dark Blue). Lyapunov exponent for each of the locked states are also shown. Shaded area show the regions where no 1∶1 locking mode is seen in numerical results, and the dashed gray lines are the boundary of stability of analytic solution with negative Lyapunov exponents. In the right panel the phase difference is shown for two HH neurons with different firing rates.

It's worth noting that in the symmetric homogeneous case when 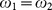, 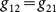 and 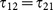, Eq. 10 reduces to 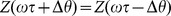 which has two solutions 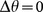 and 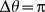. Inphase (synchronous) mode is stable if 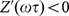 and stability condition for antiphase firing is 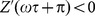. Note that the system can show bistability if both the inphase and the antiphase solutions are (locally) stable but with canonical forms of PRC (and phase sensitivity) for type-I (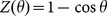) and type-II (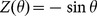) neurons, only one of the solutions are stable. Also, for some values of delay time all the phase lags are fixed points. For example, for canonical type-II neurons this occurs for 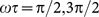. In this case the left hand side of Eq. 14 is always zero and all the phase lags are neutrally stable. In this case the initial conditions determine the phase lag in the locked state (Fig. 2a).

## Reciprocally Coupled Neurons in Presence of Inhomogeneity

### Frequency mismatch

Inhomogeneity can be exerted into the system with the mismatch in the parameters of neurons (namely their firing rate in the minimal model we used), and/or with the difference in the connections parameters, i.e., delays or synaptic strengths. In the presence of mismatch in intrinsic frequencies, with symmetric connections, i.e., 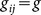, 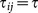 and 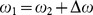, the phase difference of the two neurons in a 1∶1 phase-locked mode can be calculated from Eq. 10. We take the phase difference as 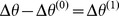, where 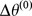 is the phase difference of the homogeneous system. For small mismatch we assume that the deviation from the phase difference of the homogeneous system, 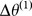, is also small and its dynamics can be described by a linearization of Eq. 12 or Eq. 13.

As an example we consider canonical type-II neurons with 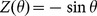. This model can describe PRC for Stuart-Landau oscillator [57] and is widely used as the canonical form of PRC near Hopf Bifurcation [58]–[61]. If the stable phase difference for homogeneous system is 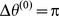 (anti-phase mode), the phase difference for the inhomogeneous system is 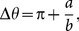
(15)

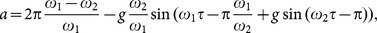


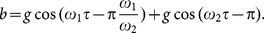



For synchronous case, 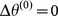, the phase lag is: 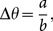
(16)

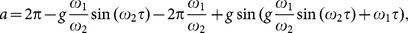


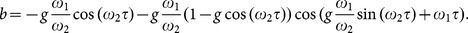



The solutions are stable if 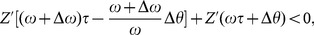
(17)for the first case and 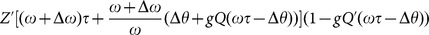


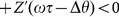
(18)for the second case. The results for canonical type-II neurons, along with the results of numerical integration of Eqs. 12 and 13 are demonstrated in Fig. 2b. Excellent accordance is seen between numerical solution of phase model and the analytical results of linear approximation. In the right panels of Fig. 2 numerical results for HH neurons have been shown, showing agreement between the results of pulse-coupled phase model with a more realistic conductance-based neuronal model.

### Inhomogious time delays and coupling constants

**Figure 3:**
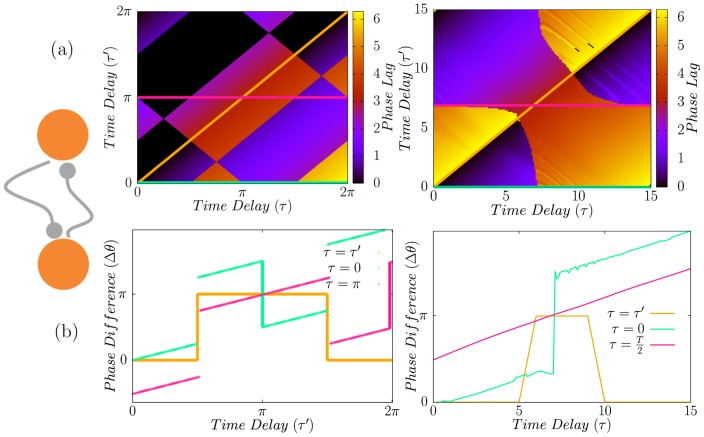
Directly coupled neurons with unequal delays. (a) Phase lag of two directly coupled neurons for different transmission time delays. In (b) we have shown the results for 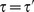 (along diagonal in (a)) and with fixed 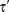 (along horizontal lines in (a)). Synchrony can only been seen for homogeneous system 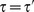. Figures in right column present the results for HH neurons. Even though the patterns are not the same, the main result still holds and synchrony can not be seen with unequal delays. 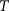 in the bottom-right panel is the period of the firing of HH neurons in the locked state.

For unequal time delays without frequency mismatch, the phase lag can be found from Eq. 10, 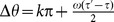 with 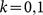, and for each value of 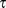 and 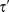 one of the two states is stable. When 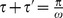, the system does not have isolated fixed points and all values of 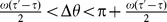 are neutrally stable solutions (Fig. 3).

**Figure 4:**
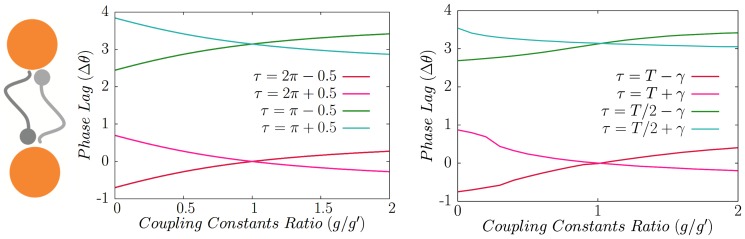
Phase locked state of two directly coupled neurons in presence of inhomogeneity in the coupling strengths. The results are shown for two different values of 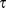 in which homogeneous network shows different properties (inphase and antiphase) in the homogenous case. The right panel show the similar result for HH neurons. Here T stands for oscillation period of HH neurons and 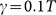.

For unequal synaptic constants 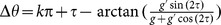, and again in presence of inhomogeneity 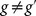 in-phase or anti-phase modes change to near inphase and near antiphase modes, respectively, and the phase lag in no longer independent of the delay time (Fig. 4).

## Dynamical Relaying

When the two neurons communicate indirectly via a third relay neuron, the symmetry of the system implies that both inphase and antiphase firing of directly connected neurons lead to synchrony of antiphase state neurons. Let 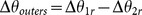, where subscript 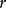 denotes the relay neuron and 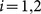 labels the outer neurons. As long as 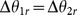, the outer neurons synchronize regardless of the value of phase difference of the outer neurons and the relay neuron.

At a steady 1∶1 locked mode, between every two successive spikes of the relay neuron, outer neurons spikes once. We construct the map by recording the phases of the three neurons at the times of spiking of the relay neuron:
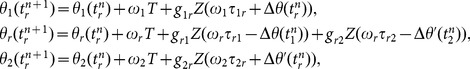
(19)where 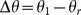, 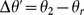.

**Figure 5:**
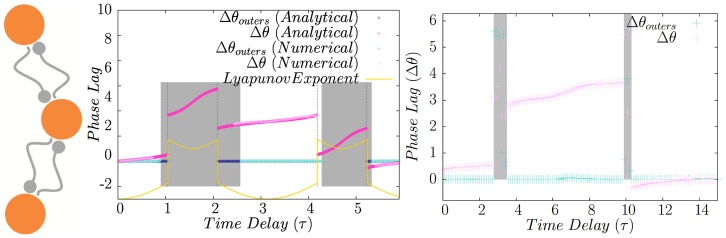
Homogeneous relay system. In a homogeneous relay system, synchronized state of outer neurons is not stable for all the values of the delay time. Dark blue and cyan points show the phase difference of outer neurons in stable regions resulted from analytic calculations and direct numerical integration, respectively. Dark pink (pink) points show analytical (numerical) results for the phase difference of the outer neurons with the relay neuron. Yellow and orange lines show the characteristic exponent of the map which is negative in case of stability of synchrony. Vertical dashed lines show the boundary of stability domains (characterized by negative exponents) and shaded area indicates the domain over which numerical integration shows no synchrony. In the right panel numerical results are presented for the relay system with Hodgkin-Huxley neurons. It is notable that although the same pattern of synchronization regions is seen, but the domain over which the synchrony is unstable is quite narrower for the relay system with HH neurons.

First we study the above equations in a homogeneous network, where 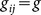, 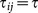 and 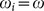. It is easy to check that the synchronous state 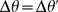 (and 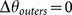) is a solution. To check the stability we note that for 1∶1 phase locked state 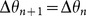, and 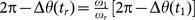. Then the equations reduce to 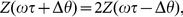
(20)and the linear stability analysis shows that the synchronous solution is stable if 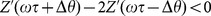. The interesting fact is that just like the two neurons system, phase lag in a homogeneous relay system motif is independent of the synaptic strength. For type-II neurons, for example, if the typical form of phase sensitivity is assumed 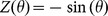, Eq. 20 gives 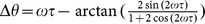. The analytic results for typical type-II neurons are plotted in Fig. 5 along with the numerical results obtained from the integration of the Eqs. 8. It can also be seen that the variation of time delay results three different stable zero time lag synchronized states for outer neurons. For 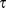 about 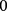 or 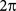 outer neurons fully synchronize, while they have small phase difference with the relay node. For the delays around 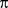 outer neurons are still fully synchronized but nearly in anti-phase mode with the relay one. The synchrony seen in the delay range for which two neurons systems shows antiphase locking is expectable as noted above, but for the three neurons system, there are intervals of time delay over which 1∶1 locked mode is not stable. These regions depicted by shaded area in Fig. 5 have a considerable measure around the 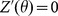 where the two neuron system have a set of neutrally stable solutions as discussed after Eq. 14. In the right panel of Fig. 5 the results are shown for a system composed of three Hodgkin-Huxley neurons. The results qualitatively conform but interestingly for HH neurons the domain of the instability of the synchronized state is much smaller, compared to the phase oscillators. Our results warrants that the domains of synchrony in the parameter space of a relay system is dependent on the model oscillators and small domain of asynchrony seen in other studies (see e.g. [43]) can be a consequence of the choice of the model oscillators (see also [40]). In the following sections we show that the domains of synchrony can be extended in some cases, by exploiting inhomogeneities which preserve the symmetry of the relay system.

### Inhomogeneity in dynamical relaying systems

Each of the three parameters, intrinsic frequency of neurons, synaptic strengths and time delays can be varied to explore the effect of the inhomogeneity in the relay system of neurons. The inhomogeneity can be exerted such that the symmetry of the system is preserved or not. For example if the firing rate of the relay neuron is different from the outer neurons, the system is inhomogeneous but symmetric, and on the other hand, if the firing rate of one the outer neurons is different from the two other neurons, the system is no longer symmetric. We hypothesize that the system is less sensitive to the inhomogeneity as long as the symmetry is not broken. [43] have shown that the synchronization of the outer neurons in the dynamical relay system is almost insensitive to the firing rate of the relay neuron. In the following we test the effect of inhomogeneity on the synchrony of the outer neurons by changing the three parameters, firing rates, transmission delays and synaptic strengths. For each of the noted parameters we test two cases, when the system is heterogeneous but symmetric and when imposed inhomogeneity breaks symmetry.

### Symmetric frequency mismatch

As the first inhomogeneous case, we suppose that the intrinsic firing rate of the relay neuron is different from the outer neurons. The set of the parameters used are such that 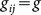, 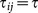, 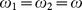 and 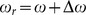. Permutation symmetry 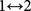 exerts that the synchronous state 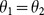 is a solution. Denoting the phase difference of the outer neurons with the relay neuron by 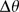, reduction of Eqs. 19 in the synchronous state 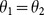 gives 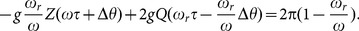
(21)


**Figure 6:**
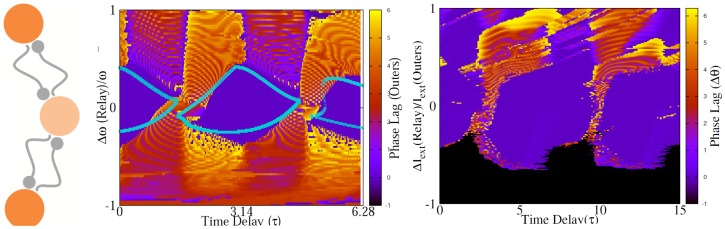
Symmetric relay system with inhomogeneous firing rates. Synchronization of the outer neurons when the relay neuron has different firing rate. In the right figure the analytic result for the borders of stable synchrony for phase oscillators is shown with blue thick lines and the numerical results for the phase lag are presented by the color code. Vertical and horizontal axis show the relative difference of the firing rate of the relay neuron (with outer neurons) and the delay time, respectively. Zero lag synchrony is coded by blue. In the right panel the numeric results are shown for HH neurons. In this case inhomogeneity is applied by changing the input current (which controls the inter-spike-interval of the neuron).

Synchronized state is stable if 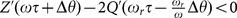. Note that the stability is still independent of the synaptic strength. In Fig. 6 the numerical result for the phase difference of the outer neuron is shown when the delay time and the frequency mismatch (between the relay neuron and the outer neurons) are varied. The analytical result for the borders of stable region shown by the solid lines matches with the region of zero phase lag (coded by blue), resulted from numerical integration. The figure shows that the tolerance of the synchrony to the mismatch of the relay neuron depends on the delay time.

For comparison we have also presented the results for a similar system composed of HH neurons in Fig. 6. It can be seen that the analytic results for phase oscillators hold qualitatively in the more biologically inspired model. It's worth noting that for HH neurons, for very small value of 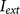 (of the relay neuron), the relay neuron does not spike and effectively the connection between two outer neurons is cut and consequently, no entrainment is expected.

### Asymmetric frequency mismatch

**Figure 7:**
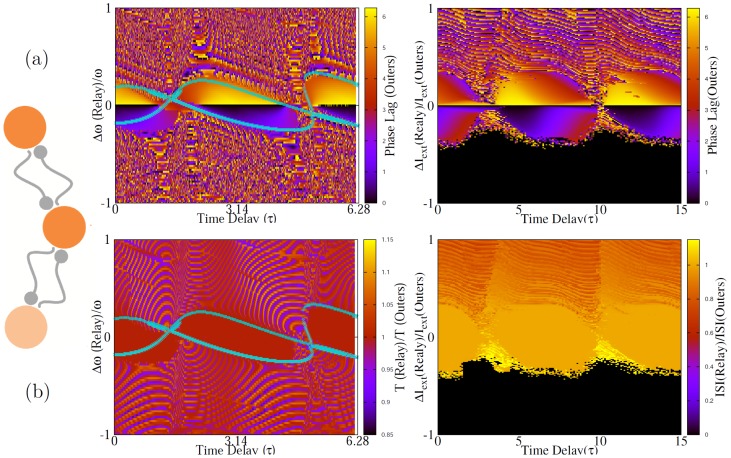
Asymmetric relay system with inhomogeneous firing rates. When frequency mismatch is applied to one of the outer neurons, no synchronized state is seen, but for some range of mismatch 1∶1 phase-locked states occur. (a) and (b) show the phase lag and ratio of the periods of outer neurons in the steady state, respectively. Blue lines are borders of 1∶1 modes from analytic stability test. In the right, same results are shown for HH neurons.

When the frequency of the outer neurons does not match (symmetry broken), 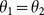 is not anymore a solution of the Eq. 21 and the outers do not spike simultaneously. This is evident from Fig. 7a which shows that phase lag of the outer neurons continuously changes with the mismatch and only for zero mismatch zero lag synchrony can be observed. In this case instead of zero lag solution, we look for a phase locked state in which 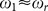. Eqs. 19 then can be written as 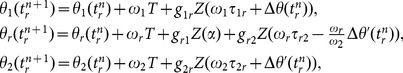
(22)where 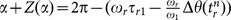. In Fig. 7b the ratio of the firing rates of the neurons is also plotted. Domain of stability of the phase-locked state (characterized by 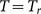) is shown by solid lines which well matches again with the numerical results. The results are supported by the numerical experiments on the HH neurons shown in Fig. 7, where a qualitatively similar dependence on the mismatch of the outer neurons can be observed.

### Different synaptic constants: Symmetric case

**Figure 8:**
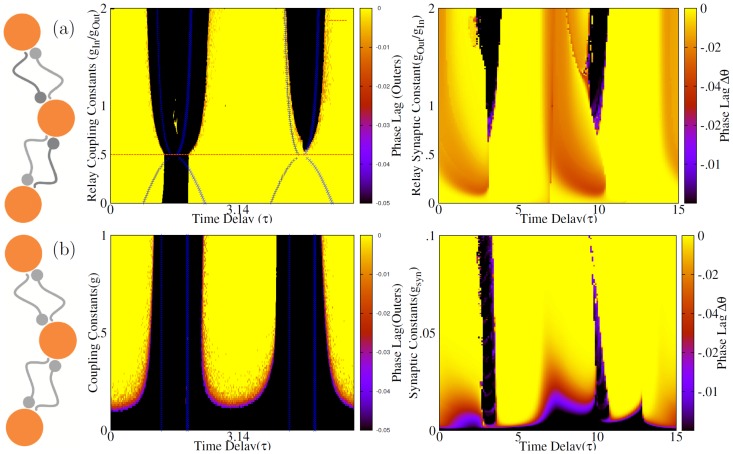
Symmetric variation of synaptic constants. (a) Synaptic constants of incoming links to the relay neuron are changed while outgoing ones are kept constant (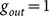). Color code shows time lag of spiking of outer neurons, resulted from numerical experiments. Solid lines are drawn based on the analytic results, showing the domain of stability of synchronous state. Note that for 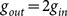 synchrony is seen for almost all the values of delay time. For comparison the results for the homogeneous system are shown in (b) where all the synaptic constants are equal. In this case except for very small synaptic constants, the results are insensitive to the changes of synaptic strengths and can not results a synchronous state. Right panel show the similar results for HH neurons.

Suppose that the strength of incoming 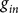 and outgoing 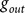 synapses to the relay neuron are different, but the counterparts on the two wings are equal. The system is symmetric and the zero lag solution 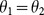 exists. Then from Eq. 19 the phase difference of the relay neuron and the outer neurons can be extracted from 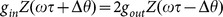, and the state is stable when 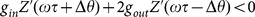. Note that the ratio of the (incoming and outgoing) synaptic constants determines both the phase difference and the stability condition in this case of symmetric unequal synaptic strengths. It is quite interesting that for 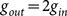 (outgoing synapses are twice as strong as the incomings), the results reduce to those of two identical oscillators, coupled by symmetric connections (see Eq. 10). In this case the equations are again independent of the connection strengths (while the ratio is preserved) and the domain of stable synchronous solution extends to all values of time delay (Fig. 8a). This can be proposed as the best configuration for zero lag synchrony of the outer neurons, since the synchrony is stable for all the values of the delay time. Enhancing effect of inhomogeneity on synchrony has been reported before for systems composed of chaotic oscillators [62]–[64]. The results shown in Fig. 8 for HH neurons indicate that this result holds for different types of neuronal models.

### Different synaptic constants: Asymmetric case

**Figure 9:**
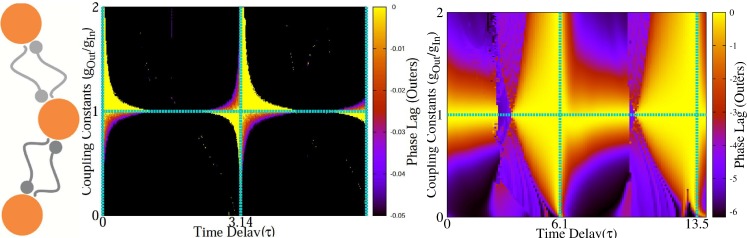
Asymmetric variation of synaptic constants. The time lag of firing of the neurons is shown as a function of delay time and ratio of synaptic constants in an asymmetrix case. Numerical results confirm outcome of analytic calculations for the phase lag of phase oscillators of type-II (left panel). Right panel shows the results for HH neurons. Again zero-lag synchrony is seen for 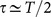 and 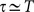.

If the connections in both sides are not of the same strengths, according to Eq. 19 synchronized state for outer neurons, 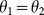, is not in general a solution of the defined map (Eqs. 19). In the simplest case when the synapses in one side are of the same strength, but they are slightly different from the synapses in other side, near synchronous states are possible. Numerical results shown in Fig. 9, are presented for typical type-II oscillators. Near synchronous results are seen when 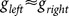 or 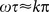.

### Inhomogeneous delays: Symmetric case

In the case of unequal transmission delays, if the symmetry is conserved, i.e., the incoming and outgoing delays (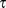 and 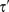) are unequal but the corresponding links in two sides have equal delays, zero-lag synchrony is still possible. Imposing 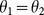 in Eqs. 19, fixed point of the map are determined by 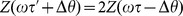 and stability condition is 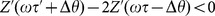. For typical type-II oscillators the phase lag of the outer neurons with the relay neuron is 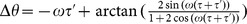.

**Figure 10:**
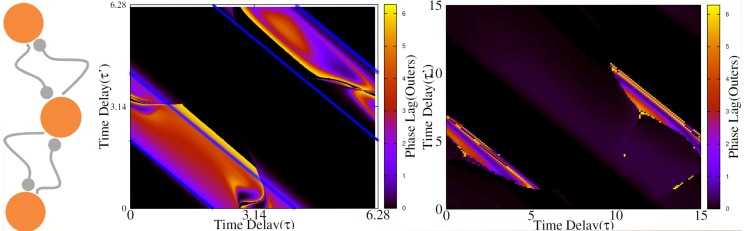
Symmetric variation of the delay times. In the left panel the phase lag of outer neurons in a relay system with canonical type-II oscillators. Horizontal and vertical axes show the incoming and outgoing delay times, respectively. Solid lines show the boundary of stable synchrony. The similar results are shown for HH neurons in the right.

In Fig. 10 we have shown the numerical results for canonical type-II oscillators. The phase lag of the outer neurons for different values of incoming and outgoing transmission delays is shown. Solid lines show the boundary of area over which the stability criterion is met. In the right panel of Fig. 10, the results for HH neurons are shown. The system behaves qualitatively the same as the type-II oscillators but there are regions in which instead of zero phase lag, very small phase lag is recorded and interestingly for small amount of outgoing synaptic time delay, complete synchrony is seen for HH neurons, regardless of the value of the incoming synaptic delays.

### Asymmetric delays

**Figure 11:**
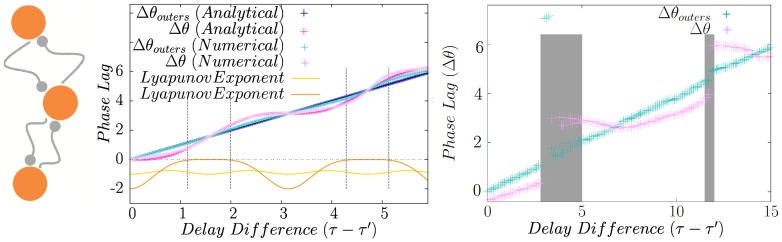
Asymmetric variation of delays. The time lag of firing of the neurons is shown as a function of difference in delay times. Numerical results confirm outcome of analytic calculations for the phase lag. Lyapunov exponents are also shown. They must be both negative for a stable phase-locked mode. In the right figure similar results are shown for HH neurons.

We assume that the delays in two wings are unequal, i.e., 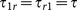 and 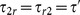. Other parameters (firing rates and synaptic constants) are assumed homogeneous. As the other asymmetric cases discussed above, zero-lag firing of the outer neurons is not possible since 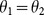 is not a solution of the Eq. 22 and in general the phase lag of the outer neurons will be a function of time delays as follows. To explore the problem analytically we consider canonical type-II oscillators. Eq. 22 results 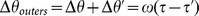 and 
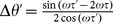
. These solutions are stable when 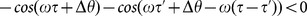 and 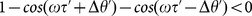, simultaneously. As depicted in Fig. 11 no zero lag synchrony occurs in asymmetric relay networks and the phase lag of outer neurons depends on difference of delays of the links in the two sides. These results are in accordance with those previously reported in [43].

I passing it's worth to ask if there are other range of parameters, other than those preserve symmetry, which result in the inphase firing of the outer neurons. We note that in the relay system (Eqs. 19), the dynamics of the relay neuron is determined by the sum of the inputs from the two outer neurons, so it can be deduced that the symmetry of the incoming links is not necessary for the synchrony of outer neurons. So as a more general result, the inphase firing of the outer neurons is possible when the outer neurons are identical and the outgoing links are of the same parameters. An example of synchrony of outer neurons with asymmetric delays of incoming connections has been reported recently [42].

## Discussion

In this study we investigated the effect of inhomogeneity on the synchronization of two neurons which communicate indirectly through a third relay neuron. This structure has been proposed as a mechanism for the synchronization of distant areas in the brain which show zero-lag synchrony despite the considerable delay in their communication [39], [41].

There are several experimental evidence that the gamma oscillations in widely separated brain areas show near zero-lag synchrony [10], [65]–[68]. Beyond the functional relevance this result is remarkable since it is not clear how the neurons can synchronize despite to the considerable delays due to axonal conduction and synaptic transmission. Many theoretical and numerical studies have devoted to the investigation of the condition for the synchronization of delayed coupled oscillators [28], [29], [33], [59], [69]. It is now well-known that the synchronization of directly coupled oscillators is dependent on their type of excitability and depending on the phase response curve and synchrony is possible for some range of delay time [33], [56], [70]. Interestingly, for a relay system the synchrony is feasible for much broader range of delay time, independent of the type of excitability of the neurons [39], [41].

The most important requirement for zero phase lag synchronization is that the relay population of cells occupies a temporally equidistant location from the pools of neurons to be synchronized [41]. This reasonable argument has been posed in our present study as a more general question. How the inhomogeneity affects the synchrony of the outer neurons in a relay system? In a minimal relay system, the three neuronal oscillators with given PRCs are characterized by their firing rate, and the connections by the synaptic strengths and the delay times. Variation of each of these parameters can be a source of inhomogeneity. Regarding to the criterion expressed above, for instance, the robustness of the system can be checked against the difference between the delay times for the incoming and outgoing synapses, when the counterparts of two wings are of equal delay. This is an example in which the inhomogeneity does not break the structural symmetry of the system. We have shown that such *symmetric inhomogeneities* has minor effect on the synchrony of the outer neurons and interestingly, in some cases, it can even enhance synchrony. Specifically, when the incoming synapses are weaker than the outgoings, synchrony is seen in a wider range of delays.

We have also shown that the relay system is not robust against *asymmetric inhomogeneity*. For example if the outer neurons have different intrinsic firing rates, their phase lag would have a finite value which is an increasing function of the mismatch between the intrinsic firing rates. In a linear approximation we have given the phase lags of the spiking of the outer neurons as a function of mismatch between the parameters of two wings, i.e., firing rates, synaptic strengths and delay times. Our results suggest that in a real relay system where neither the neuronal nor the synaptic parameters are fine tuned, near zero-lag synchrony can be expected instead of exact synchrony. In the nervous system, the firing rates are not constant and synaptic efficacies change due to the short and long term plasticities. Neuronal and synaptic changes in short time scales can lead to appearance of transient synchrony reported in different experiments on sensory systems [71]–[73]. Long term changes in synaptic strengths can also affect the collective properties of a neuronal networks [see e.g., [74]–[76] and further studies are needed to reveal the role of synaptic plsticity in the dynamics of the relay system.
